# Insights into the Genes Involved in ABA Biosynthesis and Perception during Development and Ripening of the Chilean Strawberry Fruit

**DOI:** 10.3390/ijms24108531

**Published:** 2023-05-10

**Authors:** María A. Moya-León, Yazmina Stappung, Elena Mattus-Araya, Raúl Herrera

**Affiliations:** Laboratorio de Fisiología Vegetal y Genética Molecular, Instituto de Ciencias Biológicas, Universidad de Talca, Talca 3465548, Chile; ystappung@utalca.cl (Y.S.); elena.mattus@utalca.cl (E.M.-A.); raherre@utalca.cl (R.H.)

**Keywords:** ABA biosynthesis, ABA perception, *Fragaria chiloensis*, fruit ripening, NCED, PYR/PYL receptors, phylogenic analysis, RT-qPCR

## Abstract

Hormones act as master ripening regulators. In non-climacteric fruit, ABA plays a key role in ripening. Recently, we confirmed in *Fragaria chiloensis* fruit that in response to ABA treatment the fruit induces ripening-associated changes such as softening and color development. In consequence of these phenotypic changes, transcriptional variations associated with cell wall disassembly and anthocyanins biosynthesis were reported. As ABA stimulates the ripening of *F. chiloensis* fruit, the molecular network involved in ABA metabolism was analyzed. Therefore, the expression level of genes involved in ABA biosynthesis and ABA perception was quantified during the development of the fruit. Four *NCED*/*CCDs* and six *PYR*/*PYLs* family members were identified in *F. chiloensis*. Bioinformatics analyses confirmed the existence of key domains related to functional properties. Through RT-qPCR analyses, the level of transcripts was quantified. *FcNCED1* codifies a protein that displays crucial functional domains, and the level of transcripts increases as the fruit develops and ripens, in parallel with the increment in ABA. In addition, *FcPYL4* codifies for a functional ABA receptor, and its expression follows an incremental pattern during ripening. The study concludes that *FcNCED1* is involved in ABA biosynthesis; meanwhile, *FcPYL4* participates in ABA perception during the ripening of *F. chiloensis* fruit.

## 1. Introduction

Chilean strawberry (*Fragaria chiloensis* (L.) Mill. subsp. *chiloensis* Staudt) is a native wild species from Chile that produces a fruit which is highly appreciated for its good organoleptic qualities such as aroma and taste, its exotic white color and remarkable biotic stress tolerance [1,2,3]. Albeit those extraordinary properties, the fruit softens very fast and intensively during ripening which reduces its post-harvest shelf life.

The Chilean strawberry fruit ripens with a non-climacteric pattern. Ripening is a complex process, during which a fruit develops into a fleshy, colorful and tasty fruit. For these biochemical and physiological changes, a series of metabolic pathways are switched on, such as the degradation of chlorophyll and starch, biosynthesis of pigments and volatile compounds and accumulation of sugars and organic acids [4]. It is important to highlight that the perfect coordination of those metabolic pathways is needed to develop a fruit of good quality [5]. In climacteric fruits, most of those metabolic pathways are regulated and coordinated by the hormone ethylene; however, in non-climacteric fruit ethylene does not have the same effect [5]. Several hormones have been analyzed as master ripening regulators in non-climacteric fruit, including abscisic acid (ABA) and auxins.

Hormones play essential roles in the control and coordination of several physiological events, including fruit ripening. In strawberry, hormonal changes include the rise of ABA levels along the ripening development, and this increment coincides with the drop of auxins [6]. Several reports have shown that ABA promotes the ripening of strawberry fruit. It has been documented in different species of *Fragaria* that the treatment of turning ripe strawberry fruits with ABA promotes a full ripe phenotype [7,8,9,10,11]. Treatments of fruit with ABA at the green stage and still attached to the plant additionally promotes ripening [12]. On the other hand, auxins seem to delay the ripening of non-climacteric fruit. This has been proven by the exogenous application of auxins that delays the ripening development of strawberry fruit and by the removal of the endogenous source of auxins (the achenes) that promotes the ripening of *Fragaria x ananassa* fruit [13]. Recently, it has been shown in *F. vesca* that even if auxins levels are relatively low and stable in advanced ripening fruit stages, the expression of genes related to auxin signal transduction and down-stream responses are reduced in receptacles as ABA increases [14]. Therefore, although the ABA/auxins ratio has been proposed as the coordination of fruit development and ripening in strawberry fruit [5,9], new evidence suggests that ABA could be the master regulator hormone and a key controller of gene expression during strawberry ripening [10,11].

ABA is a sesquiterpenoid hormone synthesized through the carotenoid pathway, additionally known as the ‘indirect pathway’ [15]. This pathway, widely reviewed by [16,17], initiates in plasmids with the conversion of zeaxanthin into trans-violaxanthin, a C40 precursor, by zeaxanthin epoxidase (ZEP); at this point, the pathway diverges in two ways, leading to the conversion of 9′-cis-neoxanthin or 9′-cis-violaxanthin, which are converted into xanthoxin (C15 intermediate) through an oxidative cleavage by the 9-cis-epoxycarotenoid dioxygenase (NCED); after this, an alcohol dehydrogenase at cytosol converts xanthoxin into abscisic aldehyde; finally, the abscisic aldehyde is oxidized to ABA by an abscisic aldehyde oxidase (AAO). On the other hand, ABA catabolism is mediated by CYP707A, a cytochrome P450 monooxygenase that converts ABA into phaseic acid (PA) through a catalytic hydroxylation [18]. The second form of catabolism is the conjugation of ABA by a UDP-glucosyltransferase (UGT) to form ABA–glucose ester (ABA–GE), which is an inactive form of ABA that is stored in vacuoles and the endoplasmic reticulum [19,20]. ABA–GE can be reversibly transformed into ABA by β-glucosidases and released from the endoplasmic reticulum and vacuole [21]. Therefore, ABA levels in the fruit are controlled through the balance between its biosynthesis (*NCED*) and its catabolism (*CYP707A*) [22].

ABA cellular responses are mediated by a group of soluble proteins named pyrabactin-resistant (PYR) and PYR-like (PYL) receptors (reviewed by [15]). The activation of these receptors by ABA induces the formation of a complex with the protein phosphatase 2C (PP2C), and in consequence, PP2C releases SNF1-related kinases (SnRK). After this, SnRK can phosphorylate targets such as transcription factors, ion channels, a Raf-like MAPKKKs signaling pathway and other mediators of ABA response (reviewed by [23]). It has been described that PYLs such as AtPYL4–6 and AtPYL8–10 act as monomers and have higher ABA binding affinity, interacting with PP2Cs in an ABA-enhanced manner; meanwhile, PYLs such as AtPYR1 and AtPYL1–2 act as dimers and have lower ABA binding affinity, interacting with PP2Cs in an ABA-dependent manner [24].

ABA homeostasis and ABA perception have been mainly investigated in *F. x ananassa* and *Fragaria vesca.* The key role of NCED on ABA biosynthesis in strawberry fruit was demonstrated on transgenic *FaNCED1* RNAi fruit [12] that provided transgenic fruit with reduced ABA levels compared to control fruit. In addition, the fruit remained uncolored; however, the exogenous application of ABA recovered the normal red color of the fruit. During the development of strawberry fruit, the expression of *FaNCED1* and *FaNCED2* rises as the levels of ABA increases; meanwhile, the expression of *FaCYP707A1* increases from green to white fruit, then decreases until the final stages of ripening [25]. On the other hand, nine members of the *FaPYR*/*PYL* gene family have been identified in *F. x ananassa*, and reports indicate that *FaPYL2* may play a major role in ripening [26]. In *F. vesca,* ABA homoeostasis involves the regulation of ABA catabolism and biosynthesis by feedback and feedforward loops which are linked to the repression of *CYP707A* expression and promotion of *NCED* expression at the onset of ripening [27]. In addition, the transient silencing of *FveCYP707A4a*, that increases the expression of *FveNCED5* and raises the levels of ABA, induces the expression of genes related to fruit softening (*FvePL*) [27]. *FvePYL2* transcripts reached a relatively high expression level at the red fruit stage, whereas other *FvePYLs* have diversified expression patterns [14].

In *F. chiloensis,* several pieces of evidence confirm that ABA is involved in its ripening as it induces the transcriptional changes required for fruit softening and color development [5,10,11,28,29,30]. As ABA seems to have key participation in the ripening stimulation of *F. chiloensis* fruit, the molecular network involved in ABA metabolism in ripening fruit was analyzed. Currently, there is little evidence of the participation of *NCEDs* on *F. chiloensis* fruit [11]; however, there is none for *PYR*/*PYLs*. Additionally, these genes belong to a gene family, and several members could or could not be involved in the process. Therefore, the clarification of which gene family member takes part during fruit ripening will precisely determine the critical players for softening and color development. Hence, the expression level of genes involved in ABA biosynthesis and ABA perception was quantified in *F. chiloensis* fruit as a way to provide further molecular evidence of the participation of ABA on the ripening development of this fruit. As several *NCEDs* and *PYR*/*PYLs* family members have been identified in the fruit species yet only a few of them seem to participate during fruit ripening, this work aims to identify those members involved in the ripening of this non-climacteric fruit.

## 2. Results

### 2.1. Identification of FcNCED/CCDs Family Genes and Analysis of Deduced Amino Acid Sequences

Through the annotation process by different databases, it was possible to identify two *NCEDs* and two *CCDs* sequences (*FcNCED1*, *FcNCED3*, *FcCCD1* and *FcCCD4*) into the *F. chiloensis* transcriptome (Table 1). These sequences were named according to their closest relative sequence within the *F. vesca* genome.

**Figure 1:**
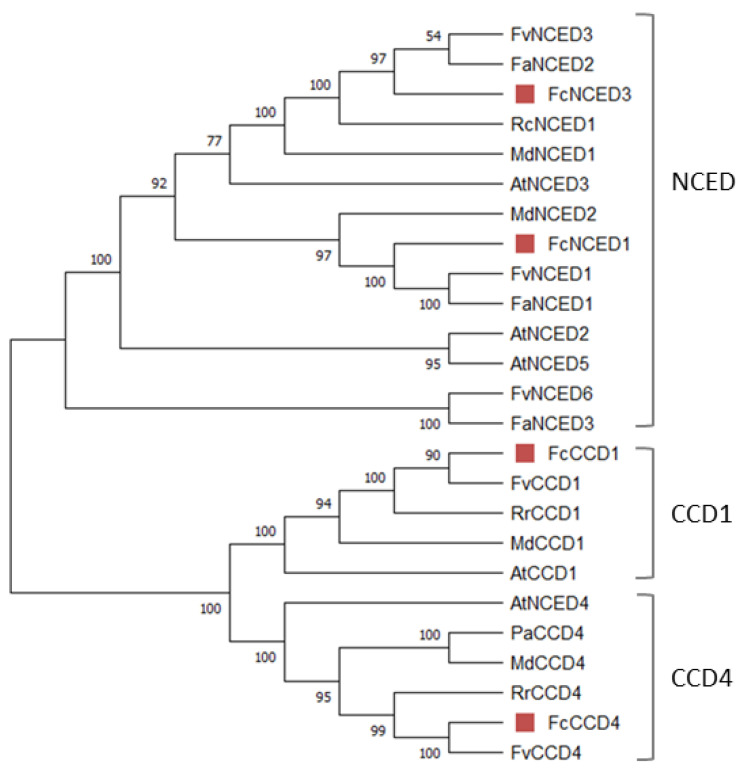
Phylogenetic analysis of FcNCEDs and FcCCDs proteins. Different *F. chiloensis* sequences (4) were aligned with 21 sequences from *F. x ananassa* (3), *M. domestica* (4), *R. chinensis* (1), *R. rugosa* (2), *P. avium* (1), *F. vesca* (5) and *A. thaliana* (5). The phylogenic tree was built using the neighbor-joining method with a bootstrap consensus tree inferred from 5000 replicates. Bootstrap values are indicated in the figure; values over 50 indicate clade robustness. The list of protein sequences employed in the analysis, including Genbank accession numbers, is shown in Table S1.

The analysis of their coding sequences recognized the characteristic domain of NCED/CCD proteins ‘Retinal pigment epithelial membrane protein’ (RPE65) in all sequences, the domain responsible for the binding of Fe^2+^. An evolutionary relationship study between different rosaceas species (*F. x ananassa*, *F. vesca*, *Malus domestica*, *Rosa chinensis*, *Rosa rugosa* and *Prunus avium*), *Arabidopsis thaliana* and *F. chiloensis* provided a phylogenetic tree that clearly separates NCEDs from CCDs into different subfamilies, and CCD comprises two clades (CCD1 and CCD4) (Figure 1). The translated amino acid sequences of *F. chiloensis* share a high sequence identity with *F. vesca* and *F. x ananassa*, confirming the parental relationship with this last species.

**Figure 2:**
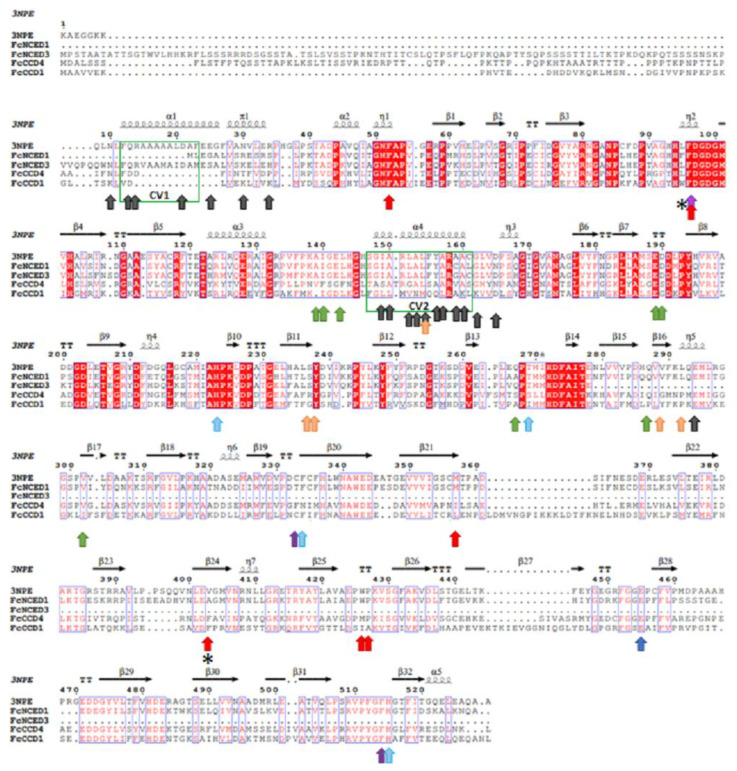
Alignment of deduced full-length amino acid sequences of FcNCED/CCD family. Amino acid sequences of FcNCED/CCDs and ZmVP14, an NCED-like protein (PDB code: 3NPE), were aligned using ESPript: gaps are indicated by dots, letters with a red background are identical amino acids and red letters are similar amino acids. The two cleavage sites (CV1–CV2) are indicated in green boxes and correspond to two antiparallel α-helix regions. Red arrows indicate the binding site for 9-cis-violaxanthin at 9-cis bond and the near methylcyclohexane; light-blue arrows indicate binding site for Fe^2+^; blue arrows indicate amino acids holding the histidines which binds Fe; green arrows indicate binding site for 9-cis-violaxanthin at isoprene chain; orange arrows indicate binding site for 9-cis-violaxanthin at methylcyclohexane; purple arrow indicates binding site for 9-cis-violaxanthin at carotenoid section between C9-C15; black arrows indicate amino acids which interact with cell membrane; and asterisks indicate amino acids that differentiate NCED and CCD (information obtained from [31]). Sequences were aligned using MAFFT version 7.

A comparative alignment was performed between the deduced protein sequences of *FcNCEDs* and *FcCCDs* and that of the crystal ZmVP14 (maize viviparous14, a key enzyme in the biosynthesis of ABA) (PDB code: 3NPE) (Figure 2) [31]. The alignment showed that the sequences share cleavage domain 2 (CV2) that interacts with 9 cis-violaxanthin and partly CV1 (Figure 2; green boxes). In addition, 2 of the 6 residues described to bind the 9-cis bond of violaxanthin and the near methylcyclohexane are highly conserved among the sequences. This is the case for the 2 conserved Phe (F52 and F96) (Figure 2; red arrows). Meanwhile, the other 4 residues vary in amino acid type; however, their hydrophobic character is maintained: Met (M357) is changed to Ile or Leu, Val (V403) for Ale or Phe, Trp (W426) for Met or Ile and finally Pro (P427) for Ala (Figure 2; red arrows). On the other hand, all the residues that coordinate Fe^2+^ (H223, H272, H337 and H515) (Figure 2; light blue arrows) and the amino acids stabilizing the His (E455) (Figure 2; blue arrows) are highly conserved between the sequences. The residues that bind the isoprene chain are also highly conserved between the sequences, except for FcCCD4. Only Glu (E189) is conserved throughout all sequences; meanwhile, replacements of Ala (A139) for Val, Ile (I140) for Phe, Leu (L143) for Phe, Asp (D190) for Ser, Met (M270) for Leu and Phe (F289) for Met are shown in FcCCD4 (Figure 2; green arrows).

There are 5 residues which have been described to bind the methylcyclohexane group, and only one of them is conserved among the FcNCED/CCD sequences: Met (M295). For the remaining four residues, high variability is detected; however, they maintain a high degree of hydrophobicity character (Figure 2; orange arrows). The residues that bind the carotenoid section between C9 and C15 are also highly conserved between the sequences, being Phe in all sequences (F97, F336 and F514) except for *FcCCD4* which in replacement of F336 there is a Met (Figure 2; purple arrow).

Importantly, two amino acid residues have been described as key to differentiate between NCED and CCD (obtained from [31]). Those residues are Leu (L95 in 3NPE crystal) and Val/Ala or Ile (V403 in 3NPE crystal) which are replaced by a Trp (W95) and a Phe (F403) in CCDs (Figure 2; asterisks). The sequences of FcNCED3 and FcCDD1 agree with bibliography; however, FcNCED1 and FcCCD4 present different expected residues in location 95. FcNCED1 presents a Phe (F95) instead of the expected Leu for NCED activity, and FcCCD4 displays a Leu (L95) instead of the expected Trp for CCD.

### 2.2. Identification of FcPYR/PYL Family Genes and Analysis of Amino Acid Sequences

The search for *PYR*/*PYL* genes within the *F. chiloensis* transcriptome allows the identification of one *PYR* and five *PYLs* sequences (*FcPYR1*, *FcPYL2*, *FcPYL4*, *FcPYL8*, *FcPYL9* and *FcPYL12*) (Table 2). The sequences were named in accordance with *F. vesca* sequences. Their coding sequences contain the conserved domain described as ‘Polyketide cyclase/dehydrase and lipid transport’, which is present in most of the sequences, except for *FcPYL2*.

**Figure 3:**
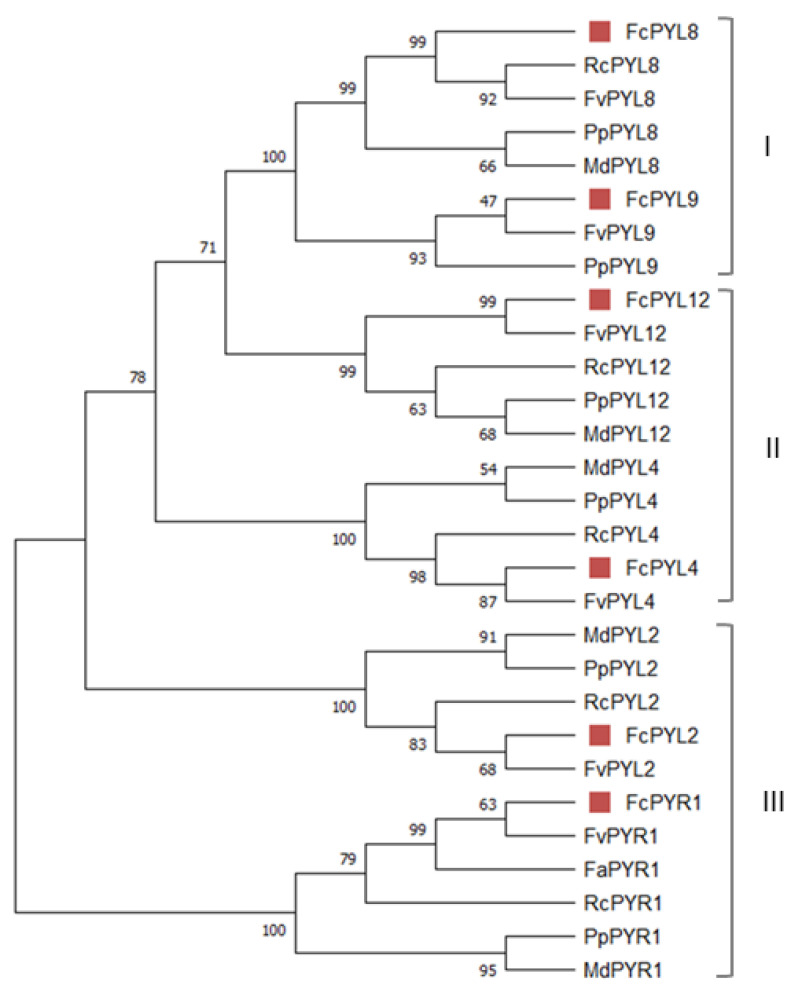
Phylogenetic analysis of *FcPYR* and *FcPYLs* proteins. Different *F. chiloensis* sequences (6) were aligned with another 23 sequences from *F. x ananassa* (1), *M. domestica* (5), *R. chinensis* (5), *Prunus persica* (6) and *F. vesca* (6). The phylogenic tree was built using the neighbor-joining method with a bootstrap consensus tree inferred from 5000 replicates. Bootstrap values are indicated in the figure; values over 50 indicate clade robustness. The sequences were grouped into three subfamilies (I–III) named as reported by [32].The list of protein sequences employed in the analysis, including Genbank accession numbers, is shown in Table S2.

The phylogenetic analysis grouped them into three subfamilies (I–III). As the nomenclature used to name each subfamily is not consistent in the literature, we decided to name them as reported by [32], based in *A. thaliana*. *FcPYR1* and *FcPYL2* belong to subfamily III; *FcPYL4* and *FcPYL12* are members of subfamily II (separated into two clades); meanwhile, *FcPYL8* and *FcPYL9* belongs to subfamily I, also known as the AtPYL8-like subfamily (Figure 3).

**Figure 4:**
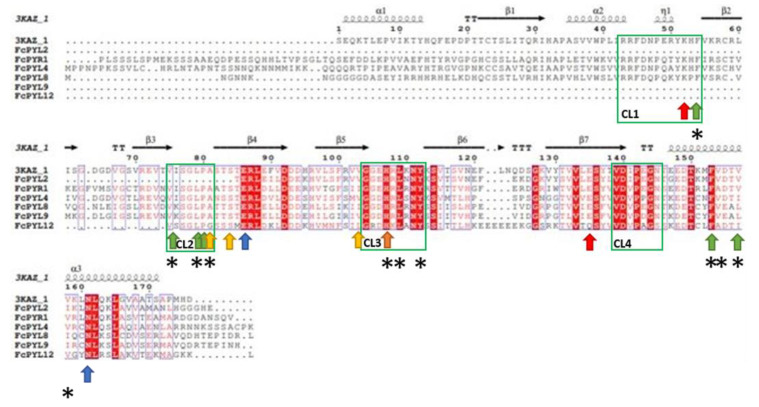
Alignment of deduced full-length amino acid sequences of FcPYR/PYL family. Amino acid sequences of FcPYR/PYLs and AtPYL2 (PDB code: 3KAZ) were aligned using ESPript: gaps are indicated by dots, letters with red background are identical amino acids and red letters are similar amino acids. The four connecting loops (CL1-CL4) are indicated in green boxes and correspond to interaction domains with ABA. Red arrows indicate the binding site for carboxyl group of ABA; blue arrows indicate hydrogen bonds with the carboxyl group of ABA; green arrows indicate binding site for methyl group of ABA; yellow arrows indicate binding site for dimethyl group of ABA; asterisks indicate amino acids present in the hydrophobic pocket; and the orange arrow indicates the binding site for PP2C protein (information captured from [33,34]). Sequences were aligned using MAFFT version 7.

A comparative alignment was performed between PYR/PYLs amino acid sequences of *F. chiloensis* and that of AtPYL2, whose 3D structure has been analyzed (PDB code: 3KAZ) (Figure 4) [33]. The alignment indicated the existence of important domains for these ABA receptors. The four connecting loops (CL1-CL4) which have been described as the interaction domains with ABA are highly conserved within the sequences, except for CL1 which is present in some of them (FcPYR1, FcPYL4 and FcPYL8) and CL2 which is absent in FcPYL2. Further, CL2 has been described as a gate and latch structure [34].

Furthermore, the sequences contain the amino acid residues involved in ABA and PP2C binding. The protein sequences coded by *FcPYR1*, *FcPYL4* and *FcPYL8* present a highly conserved Lys residue (K52) that binds to the carboxyl group of ABA (Figure 4; red arrow). All sequences possess a highly conserved Glu residue (E135) that also binds to a carboxyl group of ABA, except for *FcPYL12* which presents a Gln (Figure 4; red arrow). All sequences share 2 highly conserved residues, Glu (E86) and Asn (N161), which interact through hydrogen bonds with the carboxyl group of ABA (Figure 4; blue arrow).

An amount of 5 of the 6 residues that bind to the methyl group of ABA are highly conserved between the sequences: Phe (F54), Val (V75), Leu (L79), Pro (P80) and Phe (F153); however, the sixth residue, Val (V157), may vary for a Leu or Ile among the different *FcPYR*/*PYL* proteins (Figure 4; green arrow). Some of these residues are also involved in the hydrophobic pocket (F54, V75, L79 and F153), in addition to His (H107), Leu (L109) and Tyr (Y112) which hold a high degree of conservation; however, other residues are modified in this hydrophobic pocket, Ala (A81) for Gly, Val (V157) for Leu or Ile and Val (V158) for Ile (Figure 3; asterisk).

There are 3 residues that have been identified to bind the dimethyl group of ABA (Ala 81, Ser 84 and Val 102) (Figure 4; yellow arrow), which are mostly conserved among *F. chiloensis* sequences. Few changes exist as there is a Gly instead of Ala81 in FcPYL12, and Val102 may vary for Ile or Met. Finally, a highly conserved His among sequences (H107) allows the interaction of *PYR*/*PYL* with *PP2C* (Figure 4; orange arrow).

### 2.3. Expression Analysis of ABA Biosynthesis Related Genes in F. chiloensis

**Figure 5:**
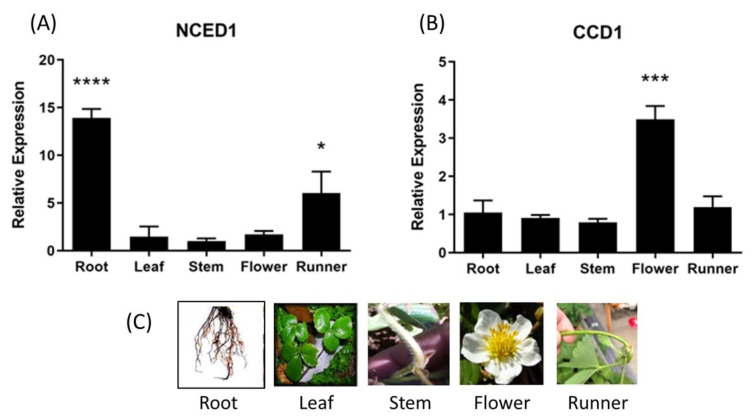
Relative expression levels of *FcNCED*/*CCDs* in several *F. chiloensis* vegetative tissues. Expression levels of *FcNCED1* (**A**) and *FcCCD1* (**B**) were determined by RT-qPCR. In (**C**) there are photographs of different *F. chiloensis* tissues. Values were first normalized against the expression data of *FcDBP* and then calibrated against stem tissue in the case of *FcNCED1* and root tissue in the case of *FcCCD1*, with a nominal value of 1. Each value corresponds to the mean ± SE of three independent RNA extractions and qPCR analysis using three technical replicates. Asterisks indicate significant differences compared to calibrated tissue (* *p* < 0.05, *** *p* < 0.001, or **** *p* <  0.0001; one-way ANOVA with Dunnet correction post hoc).

The relative expression level of ABA biosynthesis related genes, *FcNCED* and *FcCCD*, was quantified by RT-qPCR in different vegetative tissues. A high relative expression level of *FcNCED1* transcripts was noticed in roots, and a middle expression level in runners and low level in the other vegetative tissues were analyzed (Figure 5A). In the case of *FcCCD1* transcripts, a high expression level was detected in flowers and a low expression level in the other vegetative tissues (Figure 5B). On the other hand, transcripts levels for *FcNCED3* and *FcCCD4* were extremely low in the vegetative tissues under study.

**Figure 6:**
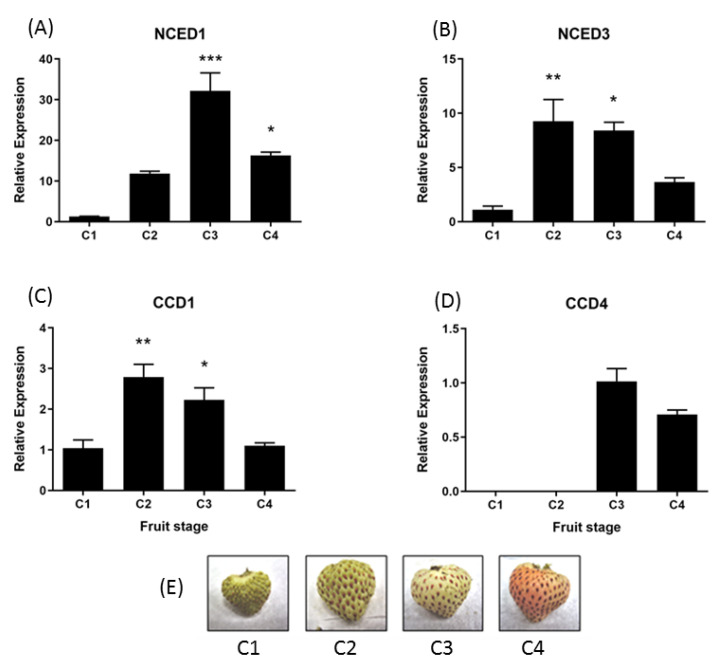
Relative expression levels of *FcNCED*/*CCDs* in *F. chiloensis* developing fruit. Expression levels were determined by RT-qPCR: (**A***) FcNCED1*, (**B**) *FcNCED3*, (**C**) *FcCCD1* and (**D**) *FcCCD4*. In (**E**) there are photographs of *F. chiloensis* fruit at different development stages. Values were first normalized against the expression data of *FcDBP* and then calibrated against the expression of C1 stage with a nominal value of 1 or C3 in the case of *FcCCD4*. Each value corresponds to the mean ± SE of three independent RNA extractions and three technical replicates. Asterisks indicate significant differences compared to C1 stage (* *p* < 0.05, ** *p* < 0.01, *** *p* < 0.001; one-way ANOVA with Dunnet correction post hoc).

In the development of fruit tissue, the relative accumulation of *FcNCED1* transcripts displays an increment during development with a maximum value at the C3 stage (Figure 6A). *FcNCED3* shows an early increment at the C2 stage which decays over time (Figure 6B). *FcCCD1* displays the same expression profile as *FcNCED3* (Figure 6C). Finally, *FcCCD4* is mainly expressed at C3 and C4 stages with no significant differences among those stages (Figure 6D).

**Figure 7:**
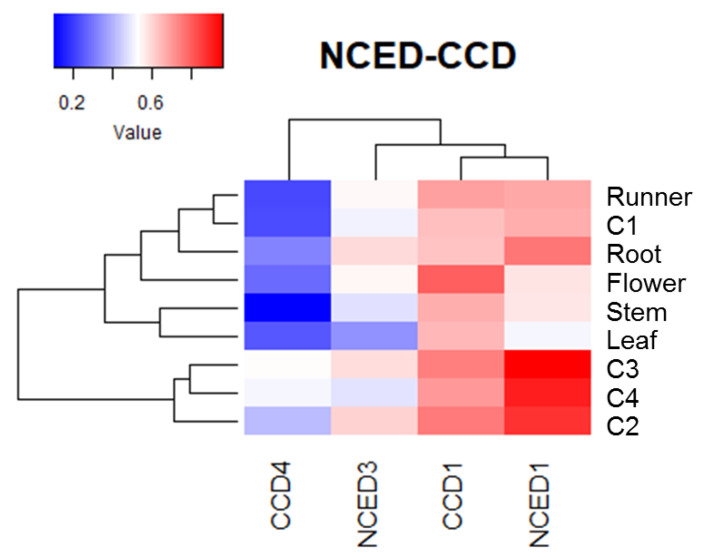
Heatmap analysis of *FcNCED*/*CCD* transcripts, clustered based on their accumulation profile and tissue specificity. The columns of the heatmap represent genes, and the rows correspond to samples. Each cell is colorized based on the expression level of a particular gene in a certain sample. The values used in the analysis correspond to the expression level of each *FcNCED*/*CCD* gene without calibration.

Heatmap analysis of non-calibrated qPCR values for ABA biosynthesis genes indicates that the expression level of *FcNCED1* was higher than for the other genes in fruit samples (Figure 7). In comparison, the expression level in fruit tissues followed the order *FcNCED1 > FcCCD1 > FcNCED3* > *FcCCD4*. In vegetative tissues, *FcNCED1* is highly expressed in roots and *FcCCD1* in flowers.

### 2.4. Expression Analysis of ABA Receptor Genes during Development of F. chiloensis Fruit and Vegetative Tissues

**Figure 8:**
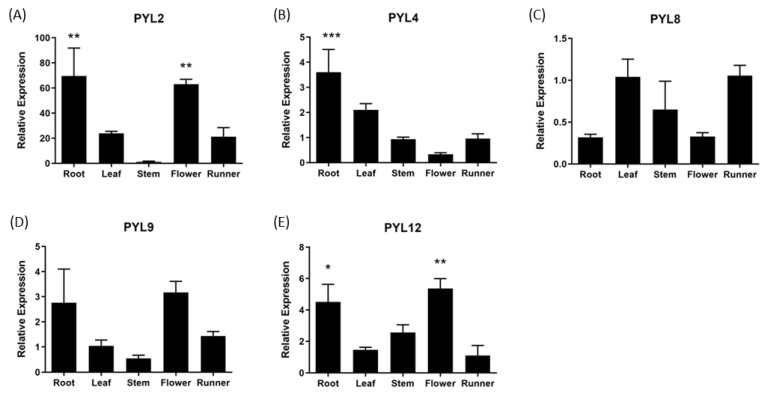
Relative expression levels of *FcPYR*/*PYLs* in several *F. chiloensis* vegetative tissues. Expression analyses were performed by RT-qPCR: Values were initially normalized against the expression data of *FcDBP* and then calibrated against a selected tissue (stem in (**A**,**B**); leaf in (**C**,**D**); and runner in (**E**)) with a nominal value of one. Each value corresponds to the mean ± SE of three independent RNA extractions and three technical replicates. Asterisks indicate significant differences compared to calibration tissue (* *p* < 0.05, ** *p* < 0.01, *** *p* < 0.001; one-way ANOVA with Dunnet correction post hoc).

**Figure 9:**
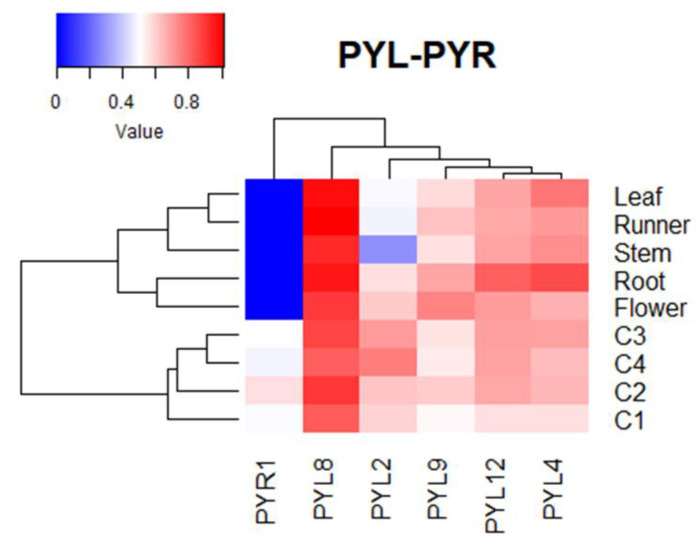
Heatmap analysis of *FcPYR*/*PYL* transcripts, clustered based on their accumulation profile and tissue specificity. The columns of the heatmap represent genes, and the rows correspond to samples. Each cell is colorized based on the expression level of each gene in a particular sample. The values used in the analysis correspond to expression values of *FcPYR*/*PYLs* without calibration.

The expression of *FcPYR*/*FcPYLs* was quantified by qPCR analysis in different vegetative tissues in order to provide a molecular evidence of ABA perception (Figure 8). Among the six different genes analyzed, only five of them were transcribed in the different vegetative tissues under analysis; an extremely low expression level was detected for *FcPYR1*. A high relative expression level of *FcPYL2* and *FcPYL12* was detected in roots and flowers and a middle to low expression in runners, leaves and stems. A relatively high level of transcripts accumulation was also observed for *FcPYL4* in roots. In the case of *FcPYL8* and *FcPYL9,* there were no significant differences in transcripts accumulation among the vegetative tissues analyzed. Heatmap analysis was built with non-calibrated expression data and indicated that the most expressed gene among vegetative tissues is *FcPYL8*, followed by a middle-high expression level in the case of *FcPYL4* and *FcPYL12* and a middle level for *FcPYL9* (Figure 9).

**Figure 10:**
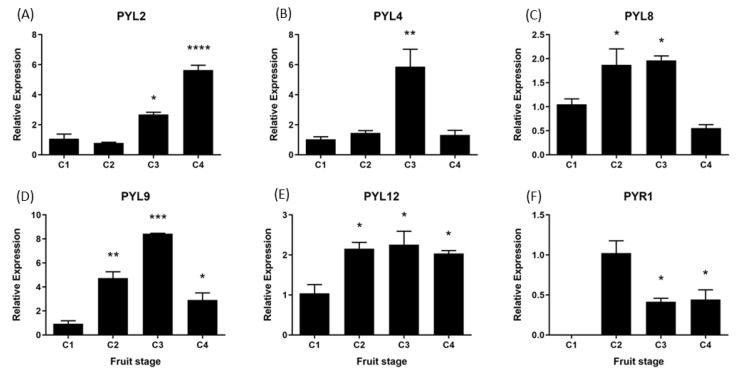
Relative expression levels of *FcPYR*/*PYL*s in *F. chiloensis* developing fruit. Expression analyses were performed by RT-qPCR: Values were first normalized against the expression data of *FcDBP* and then calibrated against the expression of C1 stage with a nominal value of 1 or C2 in the case of *FcPYR1*. Each value corresponds to the mean ± SE of three independent RNA extractions and three technical replicates. Asterisks indicate significant differences compared to C1 stage (* *p* < 0.05, ** *p* < 0.01, *** *p* < 0.001, or **** *p* <  0.0001; one-way ANOVA with Dunnet correction post hoc).

During the development and ripening of *F. chiloensis* fruit there are significant changes in the expression level of all *FcPYL*/*PYR* genes under study (Figure 10). In *FcPYL* genes, there is a clear increment in the level of transcripts along fruit development and ripening, although different profiles can be observed. In the case of *FcPYL2,* there is a clear increment in transcripts from stage 3, with maximum values at stage 4. In the case of *FcPYL4,* there is also a sudden rise at stage 3; however, it decreases after that. In the case of *FcPYL9,* the rise in transcripts is observed from stage 2, reaching the maximum value at stage 3 and then decreasing at stage 4. In the case of *FcPYL12,* a rise was observed at stage 2, and the level was maintained until the end of ripening. In the case of *FcPYR1,* a completely different expression profile was observed, with a rise in expression between stages 1 and 2, followed by a reduction at stages 3 and 4. Heatmap analysis indicated that *FcPYL8* is the most expressed gene in ripe fruit tissues, followed by *FcPYL2*, *FcPYL12*, *FcPYL4* and *FcPYL9* (Figure 10). The expression level of *FcPYR1* is very low compared to the other *PYL* genes.

### 2.5. During Fruit Development ABA Levels Correlate with ABA Biosynthesis Genes and ABA Receptor Genes

**Figure 11:**
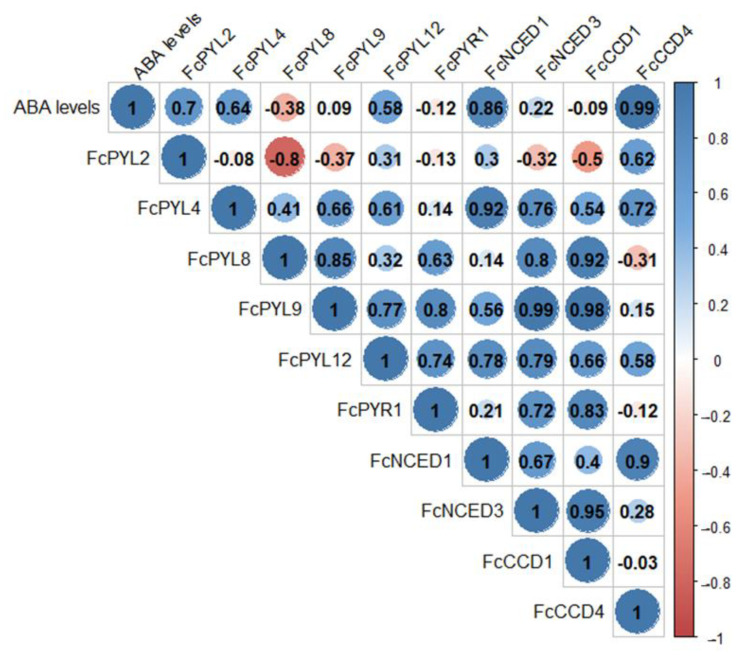
Pearson correlation analysis between ABA levels during fruit development, ABA biosynthesis genes and ABA receptor genes. Raw expression values of each gene and ABA level data from the four development and ripening stages of *F. chiloensis* fruit were analyzed in order to find correlations.

ABA levels during the development and ripening of *F. chiloensis* fruit have been reported earlier [11], showing an increment as fruit ripens, with higher levels at stages three and four. Pearson correlation values indicate a direct correlation between ABA levels during fruit development and the expression level of ABA biosynthesis genes such as *FcNCED1* (r= 0.86) and *FcCCD4* (r = 0.99) (Figure 11). This indicates that *FcNCED1* and *FcCCD4* may be involved in the biosynthesis of ABA during the ripening of *F. chiloensis* fruit. In addition, the increment in ABA is coincident with the increased expression of *FcPYL2* (r = 0.70) and FcPYL4 (r = 0.64), suggesting their participation in ABA sensing.

Positive correlations were found between the expression levels of the different genes: *FcPYL4* correlates with *FcNCED1* (r = 0.92); *FcPYL8* correlates with *FcNCED3* (r = 0.80) and *FcCCD1* (0.92); *FcPYL9* correlates with *FcNCED3* (r = 0.99) and *FcCCD1* (r = 0.98); *FcPYR1* correlates with *FcCCD1* (0.83); *FcNCED1* correlates with *FcCCD4* (0.90); and *FcNCED3* correlates with *FcCCD1* (0.95). Interestingly, the only negative correlation was found between *FcPYL2* and *FcPYL8* (r = −0.80).

## 3. Discussion

Carotenoid cleavage dioxygenases (CCDs) are a family of enzymes that catalyzes the cleavage of carotenoids into smaller apocarotenoids, molecules with relevant properties including pigments, flavor and aroma compounds and plant growth regulators such as vitamin A, ABA and strigolactone [35,36]. The *CCD* gene family comprises *CCD* and *NCED* subfamilies. Importantly, the NCED enzyme can catalyze the cleavage of the 11, 12 double bond of violaxanthin (C40) or neoxanthin (C40) to form xanthoxin (C15), the precursor of ABA. The reaction carried out by NCED is considered the rate-limiting step in ABA biosynthesis.

In *Fragaria chiloensis,* two *NCEDs* and two *CCDs* were identified within the transcriptome prepared from fruit samples [37]. In general, the number of *CCD* genes identified in the genome of different fruit species is reduced compared to the number described in non-fruit species (30 in oilseed rape; 19 in tobacco) [35]. In the genome of watermelon (*Cucumis lanatus*), melon (*Cucumis melo*) and cucumber (*Cucumis sativus*) a recent report indicated the existence of 10, 9 and 9 *NCED*/*CCD* protein sequences, respectively [35]. In all these cucurbitaceae species there are four *NCED* sequences and five or six *CCDs*. In litchi (*Litchi chinensis*), 15 *LcCCO* genes were identified in its genome, 3 *NCEDs* and 12 *CCD*s [36]. In tomato, seven *CCD* sequences have been described within its genome [38]. In comparison, in Arabidopsis a total of nine members of the *CCD* family have been reported, divided into four *CCDs* and five *NCEDs* [39]. Therefore, the number of *CCD* members in *F. chiloensis* is reduced compared to other fruit species; however, as the search has been restricted to a fruit transcriptome there may be other sequences waiting to be discovered.

Several studies suggested that different *CCD* subfamilies exhibit different roles within the plant kingdom. For example, *CCD1* plays important roles in the aroma and flavor of horticultural products as it catalyzes the formation of α-ionone, β-ionone and geranylacetone [40,41]. *CCD4* contributes to color formation in flower petals and fruit peel and additionally aroma production [42,43,44,45,46,47,48]. *CCD7* and *CCD8* seem to participate in the biosynthesis of the hormone strigolactone, which could control shoot branching, reproductive development and plant responses to drought and salt stress [49,50,51,52]. Finally, the *NCED* subfamily is involved in ABA biosynthesis and closely involved in fruit development and ripening. In the commercial strawberry, *FaNCED1* has been demonstrated to be crucial for ABA biosynthesis, as RNAi constructs promoted a dramatic reduction in ABA content that reduced the development of a red color [12], and the phenotype was reverted by the application of exogenous ABA. Similarly in grape [53], sweet cherry [54] and litchi fruit [55], ABA accelerated the accumulation of anthocyanins by increasing the expression of *NCED*.

In litchi *LcCCD4s*, *LcCCD1*, *LcNCED1* and *LcNCED2* might participate in postharvest storage of the fruit and peel coloration [36]. The expression of *LcNCED1* in fruits was consistent with the accumulation of ABA during the ripening of litchi [56]. In peach and grape fruits, *PpNCED1* and *VvNCED1* transcripts increase at the early stages of ripening, which initiates ABA biosynthesis and ABA accumulation [57]. In *Citrus clementina*, the expression of *CcNCED5* increases at color break and remains high at the ripe stage in parallel to ABA levels, suggesting a role in the ripening of mandarin fruit [58].

In melon, the expression of *CmCCD1* was upregulated during the development of the fruit and seems to participate in aroma formation [59]. In addition, *CmCCD1* was upregulated by ABA and other stress conditions such as drought and salt [35]. The expression of *CmNCED3* was upregulated in melon leaves under a series of abiotic stressors (salt, cold and drought), indicating that it plays an important role in stress [35]. Finally, *CmNCED5s* are highly expressed in flowers and play crucial roles in flower growth and development [35].

From the *FcNCED*/*CCD* sequences identified in *F. chiloensis*, all of them were detected in fruit tissues, albeit with different profiles. This is not surprising, as the sequences were obtained from a fruit transcriptome. Only *FcNCED1* and *FcCCD1* were detected in vegetative tissues: *FcNCED1* in roots and runners and *FcCCD1* mostly in flowers.

The phylogenetic analysis grouped *FcNCED1* in the same branch with *FaNCED1* and *FvNCED1*. Our data indicate that the expression level of *FcNCED1* increases as the ripening of the fruit is taking place. This also correlates with the increment in ABA observed in the fruit during development. Interestingly, this gene is mainly expressed in fruit tissues, with a low expression level in roots. All this evidence indicates that *FcNCED1* is the orthologous of *FaNCED1*, a gene which has been demonstrated to be involved in ABA biosynthesis during the ripening of *F. x ananassa* fruit whose repression by RNAi avoided the biosynthesis of ABA [12]. Importantly, the expression of *FcNCED1* has been reported to be induced in fruit by ABA treatment [11].

The alignment of deduced amino acid sequences indicated that FcNCEDs and FcCCDs share important domains related to activity. The amino acid residues identified as a part of cleavage domains are mostly conserved. Furthermore, 2 amino acids seem to discriminate between NCED and CCD proteins: L95 and V403 from ZmVP14. The corresponding residues in FcNCED3 and FcCCD1 are in agreement with a hypothetic differentiation for activity; however, in the case of FcNCED1 and FcCCD4 the residue at location 95 is not the expected. FcNCED1 contains a Phe instead of the expected Leu, and FcCCD4 contains a Leu instead of Trp. Nevertheless, the changes in amino acids do not interfere with their character, as Phe, Leu and Trp are nonpolar hydrophobic residues. Interestingly, the same Phe identified in FcNCED1 exists in FvNCED1 and AtNCED3, and in the case of FcCCD4, the Leu in position 95 also exists in FvCCD4, RrCCD4, PaCCD4 and MdCCD4. Therefore, the discrimination between these two activities requires further studies.

The search for ABA receptor sequences within the *F. chiloensis* fruit transcriptome provided the identification of one *PYR* and five *PYLs* sequences. The translated amino acid sequences displayed the connecting loops required for the interaction with ABA and most of the residues involved in ABA and PP2C binding. The phylogenetic analysis grouped them into three subfamilies that correlates well with the number of subfamilies reported in many species. As in Arabidopsis, subfamily II includes FcPYL4 in one clade and FcPYL12 in another. Interestingly, this second clade is missing in monocot species and additionally in some dicots such as tomato and citrus [32]; however, it is present in *F. chiloensis*, a Rosaceae species. The three PYR/PYL families have arisen during evolution; the latest to emerge is subfamily III, which is present only in angiosperms [40]. Subfamilies I and II are composed of monomeric receptors with high basal activity and require only a low level of ABA to induce PP2C inhibition, whereas subfamily III receptors are dimeric in solution and have low basal activity [60].

All *FcPYR*/*PYLs* were expressed in *F. chiloensis* fruit tissues, and all of them except for *FcPYR1* were expressed in vegetative tissues. *FcPYL8* was the most expressed gene in vegetative tissues. Different expression profiles were obtained for *FcPYR*/*PYLs* in ripening fruit. Of interest is the expression of *FcPYL2* and *FcPYL4* along the ripening progress, as their expression followed the increment in ABA levels reported in the fruit, suggesting their participation in ABA perception. Nevertheless, *FcPYL2* does not contain CL2 which is crucial for ABA interaction.

The expression of the PYR/PYL gene family has been analyzed during ripening in different fruit species. In cucumber, *CsPYL2* was expressed at a high level during fruit ripening, with a peak of expression between turning and ripe stages, coincident with the highest level of ABA content in the fruit, suggesting its participation in ABA perception during fruit ripening [61]. In Chinese white pear, from the 11 *PbrPYL* genes identified in its genome, 7 of them, which were distributed within the 3 subfamilies, were expressed in ripe fruit (*PbrPYL1*/*4*/*5*/*6*/*7*/*8*/*9*) [61]. In grapes (*Vitis vinifera*), from the 8 PYL genes identified in the genome, 2 of them have a particular expression profile of interest for fruit ripening, as *VvPYL1* and *VvPYL8* significantly increased from fruit set until the ripening stage [62]. Those two genes can be classified in subfamily I. In a citrus genome, 11 *PYL* sequences have been identified [32]. Expression analysis in fruit performed in only six *CsPYLs* has shown that *CsPYL9* belonging to subfamily I was the most expressed during sweet orange development and ripening [63]. In tomato, a climacteric fruit, two of the fourteen *SlPYL* genes identified displayed an expression peak at breaker stages when ripening is starting and were reported as candidates to regulate fruit ripening [64]. These two genes belong to subfamilies I and II, according to our nomenclature. A recent study confirmed that among the 14 *SlPYLs*, the expression level of *SlPYL1* was the highest, and the expression patterns of *SlPYL1*, *SlPYL4* and *SlPYL9* agreed with the ABA accumulation in fruit during development [65]. Interestingly, *SlPYL9* which is closest to monomeric *AtPYL4* and therefore belongs to subfamily II, seems to have a specific role in fruit development and ripening. The overexpression of *SlPYL9* accelerated the ripening of the fruit; meanwhile, RNAi lines showed a delay in ripening [65].

It has been proposed that *PYR*/*PYL* genes grouped into the same subfamily may perform similar functions; however, as described, members of the three subfamilies have been identified with a role during ripening in several fruit species (*F. chiloensis*, tomato, Chinese pear, grapes and citrus), and therefore, this statement is not fully true in the case of fruit ripening.

In conclusion, 2 out of the 10 genes analyzed were finally selected for being involved in ABA biosynthesis and ABA perception in *F. chiloensis* fruit: *FcNCED1* and *FcPYL4*. *FcNCED1* is expressed with an increasing pattern during its ripening, which correlates with the ABA levels reported for the fruit. In addition, the gene also increments its expression in response to ABA. On the other hand, *FcPYL4* from subfamily II displays an expression profile which may explain ABA perception. According to the findings, these two genes, *FcNCED1* and *FcPYL4*, might participate during the softening and color development of the *F. chiloensis* fruit.

## 4. Materials and Methods

### 4.1. Plant Material

White Chilean strawberry fruit (*F*. *chiloensis* (L.) Mill. subsp. *chiloensis* f. *chiloensis* Staudt) and vegetative tissues (leaves, flowers, runners, stems and roots) were collected from plants growing in a commercial field in Purén, The Araucanía Region, Chile. The plants have been propagated through stolons and correspond to the cultivated species from Purén [3]. Vegetative tissues such as leaves, runners, stems and roots were obtained from a set of five plants; meanwhile, fruits and flowers were collected from plants growing in the same field lot. The fruit was segregated into four different stages: C1, C2, C3 and C4, as recently reported [11]. An amount of 3 pools of fruit tissue were prepared, including 5 fruits from each stage. Fruit and vegetative tissue samples were frozen in liquid nitrogen, converted into a powder with the help of a mortar and pestle and then stored at −80 °C until use.

### 4.2. F. chiloensis Gene Sequences

Genes annotated as *NCED*/*CCD* and *PYR*/*PYL* were selected from the Genome Database of Rosaceae [66] using *Malus domestica*, *Prunus persica*, *Rosa chinensis* and *F. x ananassa* as reference species. Further, a comparative mapping of selected sequences and a *F. chiloensis* transcriptome was carried out as a way to identify their orthologs [37]. Sequences were aligned using MAFFT version 7 (https://mafft.cbrc.jp/alignment/server/, accessed on 7 October 2021). The search provides one ortholog sequence for *PYR*, five for *PYL*, two for *NCED* and two for *CCD*. The sequences were named based on the best alignment against the NR database.

### 4.3. Phylogenetic Analysis and Motif Analysis

Phylogenetic analyses were performed to analyze the evolutionary relationship of NCED–CCD and PYR–PYL sequences. The analyses were conducted by MEGA software v10.1. Sequence alignment was performed through the CLUSTAL W method and the phylogenetic tree by the neighbor-joining algorithm with 5000 bootstrap replicates.

The amino acid sequences deduced from *F. chiloensis* genes were analyzed using Pfam prediction, searching for conserved evolutionary domains related to ABA.

### 4.4. RNA Extraction and Expression Analysis by Real Time PCR (qPCR)

The procedure described in [11] was employed to extract RNA and to perform cDNA synthesis from fruit/tissue samples. Specific primers for qPCR analysis are listed in Table 3. Further, qPCR reactions were carried out in triplicates and employing three independent cDNA preparations from each biological sample. Relative expression levels correspond to the mean of three biological replicates ± SE, normalized against the expression level of *FcDBP* (DNA binding protein) (constitutive gene) [67]. The C1 fruit stage was employed as a calibrator for fruit samples, and the tissue with the lowest expression was employed as a calibrator for the analysis of vegetative tissues. Asterisks indicate significant differences among fruit stages or different tissues.

### 4.5. Heatmap Analysis

Heatmap analysis was generated using the open-source software R—graphic interface R-Studio (1.3.1093). The diagrams and dendrograms were obtained with the packages “gplots” and “corrplot” which are included in the R library.

### 4.6. Statistical Analysis

For gene expression analysis, a random design with 3 biological replicates and 3 technical replicates was employed. For comparisons of qPCR results, one-way ANOVA was used with Dunnet correction post hoc; differences were considered statistically significant when *p* < 0.05 (*), *p* < 0.01 (**), *p* < 0.001 (***) or *p* <  0.0001 (****).

## Figures and Tables

**Table 1:** Analysis of functional domains of deduced FcNCED/CCD amino acid (aa) sequences using PFAM database.

Gene	Transcript ID	Domain Type	Seq Length (aa)
*FcNCED1*	comp919_c0_seq1	Retinal pigment epithelial membrane protein	501
*FcNCED3*	comp27198_c0_seq1	Retinal pigment epithelial membrane protein	392
*FcCCD1*	comp799_c0_seq1	Retinal pigment epithelial membrane protein	568
*FcCCD4*	comp503_c0_seq1	Retinal pigment epithelial membrane protein	581

**Table 2:** Analysis of functional domains of deduced FcPYR/PYL amino acid sequences using PFAM database.

Gene	Transcript ID	Domain Type	Seq Length (aa)
*FcPYR1*	comp13086_c0_seq1	Polyketide cyclase/dehydrase and lipid transport	216
*FcPYL2*	comp7632_c0_seq1	ND	93
*FcPYL4*	comp22550_c0_seq1	Polyketide cyclase/dehydrase and lipid transport	218
*FcPYL8*	comp2447_c0_seq3	Polyketide cyclase/dehydrase and lipid transport	189
*FcPYL9*	comp3997_c0_seq2	Polyketide cyclase/dehydrase and lipid transport	121
*FcPYL12*	comp2932_c0_seq1	Polyketide cyclase/dehydrase and lipid transport	167

ND: not determined.

**Table 3:** Nucleotide sequence of the primers used in the qPCR analyses (Tm of 60 °C).

Gene		Sequence(5′→3′)	Efficiency
*FcNCED1*	Fw	GATCTACCTTGGCGAAACCA	90.0%
	Rv	GAGGCGGATCATGTGAACTT	
*FcNCED3*	Fw	ACGACTTCGCCATTACCG	92.1%
	Rv	AGCATCGCTCGATTCT	
*FcCCD1*	Fw	GCCAAGCATATGACACTCCTC	98.0%
	Rv	TCCTCGTTAGAAGGCCTGAA	
*FcCCD4*	Fw	CATTCCCGACCAAGATAGGA	94.3%
	Rv	GCCGTCCTTTGAGTAAACC	
*FcPYL2*	Fw	GCCATGGTGGTCAACTGTTA	100.5%
	Rv	CTGGGATTCTGGGGTACAC	
*FcPYL4*	Fw	ATGCCTCCCAACCCACCCAA	104.0%
	Rv	CGCTGCTGCTGCTGCTTCTT	
*FcPYL8*	Fw	TGAAGCTTCCGAGCTTTCAT	92.1%
	Rv	GGTCCTTAAACTTTGACGGAAG	
*FcPYL9*	Fw	GATGCACCTGCTAACAAAAGG	90.1%
	Rv	CAATCGTGTTTCTCATTTGTGC	
*FcPYL11*	Fw	GAAAGGATGGCTGGTAATTGA	91.0%
	Rv	CGCTACAACAAGAAGTCAAGAA	
*FcPYL12*	Fw	TTTCCTATCCTGCTGCTGCT	96.1%
	Rv	GCTTAGAATCCGAAACGGACTA	
*FcPYR1*	Fw	GCTACCCAAATTGCTGAACC	99.3%
	Rv	GAATGACGAAAATGTCCTTGG	

## Data Availability

All data generated or analyzed during this study are included in this published article.

## Supplementary Materials

The supporting information can be downloaded at the: https://www.mdpi.com/article/10.3390/ijms24108531/s1.
