# Supplementary material for: Insights into the Genes Involved in ABA Biosynthesis and Perception during Development and Ripening of the Chilean Strawberry Fruit
Source: Int J Mol Sci. 2023 May 10;24(10):8531. doi: 10.3390/ijms24108531 (PMC10217956; doi:10.3390/ijms24108531)
Supplement: Supplementary file 1 [file ijms-24-08531-s001.zip › ijms-2372387-supplementary.pdf]

SUPPLEMENTARY MATERIAL

Table S1. List of protein sequences employed in the phylogenetic analyses of *PYR/PYL*, including GenBank accession numbers.

**Species**	**Id**	**GeneBank**
Fragaria x ananassa (Fa)	*PYR1*	ADZ55282.1
	PYR1-like	XP_008341960.1
	PYL2	XP_008377517.1
Malus domestica (*Md*)	*PYL4-like*	XP_008370174.2
	PYL8	XP_008380616.1
	PYL12-like	XP_008371472.1
	PYR1	XP_007209595.1
	PYL2	XP_007225598.2
Prunus persica (*Pp*)	*PYL4*	XP_020421077.1
	PYL8	XP_007218467.1
	PYL9	XP_007218488.1
	PYL12	XP_007213534.2
	PYR1	XP_024172755.1
	PYL2	XP_024167402.1
Rosa chinensis (*Rc*)	*PYL4*	XP_024189542.1
	PYL8	XP_024177069.1
	PYL12	XP_024160584.1
	PYR1	XP_004300241.1
	PYL2	XP_004291031.1
Fragaria vesca (*Fv*)	*PYL4*	XP_004302017.1
	PYL8	XP_011470277.1
	PYL9	XP_004306686.1
	PYL12	XP_004293952.1

Table S2. List of protein sequences employed in the phylogenetic analyses of *NCED/CCD*, including GenBank accession numbers.

Species	Id	GeneBank
Fragaria x ananassa (*Fa*)	*NCED1*	XP_004293530.1
	NCED2	AFU61915.1
	NCED3	AFU61916.1
Malus domestica (*Md*)	*NCED1*	XP_008382970.1
	NCED2	XP_008371764.2
	CCD1	XP_008375177.3
	CCD4	XP_008340019.2
Prunus avium (*Pa*)	*CCD4*	XP_021809777.1
Rosa chinensis (*Rc*)	*NCED1*	XP_024156554.1
Rosa rugosa (*Rr*)	*CCD1*	AKT74334.1
	CCD4	AKT74335.1
Fragaria vesca (*Fv*)	*NCED1*	XP_004293530.1
	NCED3	XP_004300667.1
	NCED6	XP_004289731.2
	CCD1	XP_004306539.2
	CCD4	XP_004297644.1
Arabidopsis thaliana (*At*)	*NCED2*	NP_193569.1
	NCED3	NP_188062.1
	NCED4	NP_193652.1
	NCED5	NP_174302.1
	CCD1	NP_191911.1
